# How to Measure Molecular Forces in Cells: A Guide to Evaluating Genetically-Encoded FRET-Based Tension Sensors

**DOI:** 10.1007/s12195-014-0368-1

**Published:** 2014-12-02

**Authors:** Anna-Lena Cost, Pia Ringer, Anna Chrostek-Grashoff, Carsten Grashoff

**Affiliations:** Group of Molecular Mechanotransduction, Max Planck Institute of Biochemistry, Am Klopferspitz 18, Martinsried, 82152 Germany

**Keywords:** Mechanobiology, Förster resonance energy transfer, Biosensors, Mechanotransduction, Fluorescence lifetime imaging microscopy

## Abstract

The ability of cells to sense and respond to mechanical forces is central to a wide range of biological processes and plays an important role in numerous pathologies. The molecular mechanisms underlying cellular mechanotransduction, however, have remained largely elusive because suitable methods to investigate subcellular force propagation were missing. Here, we review recent advances in the development of biosensors that allow molecular force measurements. We describe the underlying principle of currently available techniques and propose a strategy to systematically evaluate new Förster resonance energy transfer (FRET)-based biosensors.

## Introduction

Cells are exposed to a wide range of mechanical forces. Endothelial cells, for instance, are subject to high shear stress in arteries but low forces in veinous or lymphatic vessels,^14^ cardiomyocytes bear the rhythmic contractions of the heart,^27^ keratinocytes are stimulated by shear or tension in the skin,^57^ and chondrocytes sense forces from cartilage compression.^51^ Interestingly, cells respond to such mechanical stimuli—that may vary over orders of magnitude—with astonishing specificity suggesting that cell type-specific mechanisms exist, which convey fine-tuned mechanoresponses. Indeed, a range of subcellular structures mediating different aspects of mechanotransduction has been identified including mechanosensitive ion-channels,^16^ the plasma membrane,^18^ the cytoskeleton,^26^ the nucleus,^24^ and cell-adhesion complexes.^21^ Techniques such as traction force microscopy^67^ have greatly contributed to our understanding of force transduction across these subcellular structures.^3^ Yet, how forces propagate on the molecular level is still largely unknown.

## Molecular Force Transduction Occurs in the Piconewton Range

**Figure 1:**
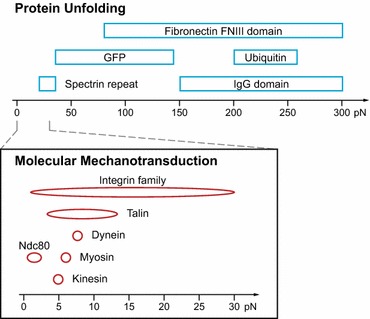
Mechanotransduction occurs in the low pN range. Motor proteins such as dynein,^22^ myosin^25^ or kinesin,^70^ cell adhesion molecules like talin^15^ or some integrins receptors^31^,^33^,^41^,^47^ as well as the kinetochore protein Ndc80^54^ are sensitive to forces below 30 pN. Even though single protein domains like spectrin repeats unfold in the similar force range,^38^,^60^ most proteins require higher forces for unfolding. GFP, for example, starts to unfold at 35 pN^17^ and even higher forces (80–300 pN) are necessary to unfold fibronectin’s FN type III domain,^50^,^59^ ubiquitin,^11^ or the IgG domain.^58^ It should be noted that some of the force ranges are still controversial and that this figure only summarizes the currently published results

A major breakthrough for our understanding of molecular force transduction has been the development of highly sensitive atomic force microscopy (AFM)^55^ as well as optical^8^ and magnetic^32^ tweezer systems, which allow researchers to scrutinize mechanical responses of single molecules *in vitro*.^81^ Such experiments revealed that forces produced by microtubule-binding motor proteins, such as kinesins or dyneins, are in the range of 5–7 piconewton (pN) per molecule^22^,^70^ (Fig. 1), highly similar to forces generated by growing microtubules (3–4 pN)^19^ or f-actin–binding myosin motors (3–4 pN).^20^

The notion that mechanotransduction—the translation of mechanical information into a biochemical response—may occur at similar forces was supported by the observation that conformational changes in the adhesion protein talin can be induced by mechanical tension as low as 2 pN.^15^,^77^ Likewise, cleavage of the von Willebrand factor is facilitated by force-induced protein unfolding at 5 pN^80^ and collagen proteolysis is increased 100-fold upon application of 10 pN force.^1^ Interestingly, some receptor-ligand pairs form adhesive interactions, called catch bonds, which strengthen under pN forces.^72^ The linkage between P-selectin and monomeric P-selectin glycoprotein ligand-1, for instance, is characterized by catch bond behavior below 11 pN,^42^,^71^ and α5β1 integrin shows enhanced binding to fibronectin type III repeats at 10–30 pN.^33^ As the unfolding of whole protein domains usually requires higher forces—the green fluorescent protein (GFP) starts to unfold at about 35 pN^17^ and immunoglobulin (Ig) or fibronectin type III domains unfold at 80–300 pN^50^,^58^,^59^—it appears that important aspects of mechanotransduction do indeed occur in the low pN range (Fig. 1). But how can such low forces be measured in cells?

## Current Approaches for Measuring Molecular Tension

**Figure 2:**
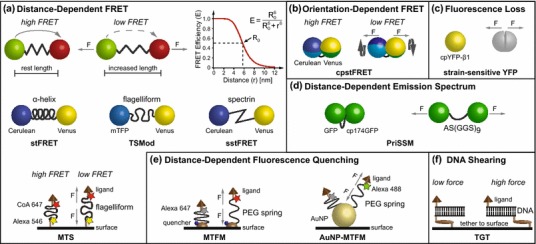
Overview of existing tension sensing techniques. (a) Distance-dependent FRET-based tension sensor modules use elastic linker elements that extend in response to force (*F*). Sufficient length increase of the linker under force is critical as the FRET efficiency (*E*) is highly dependent on the chromophore separation distance (*r*). Currently available FRET pairs are characterized by Förster distances (*R*_0_) of 5–6 nm; as an example, the FRET vs. distance correlation for *R*_0_ = 5.8 nm is shown. Employed linkers include an α-helix in strain-sensitive FRET (stFRET),^46^ (GPGGA)_8_ repeats in the flagelliform tension sensor module (TSMod)^23^ as well as in the molecular tension sensor (MTS),^47^ and a spectrin repeat in spectrin stFRET (sstFRET).^43^ (b) Force across the circularly permuted (cp) stFRET (cpstFRET) sensor rotates the fluorophores thereby reducing FRET efficiency.^44^ (c) Force across a strain-sensitive cpYFP causes fluorescence loss.^28^ (d) In the proximity imaging-based strain sensor module (PriSSM), the emission spectrum changes in response to force-dependent distance increase between green fluorescent protein (GFP) and cp174GFP.^29^ (e) Some tension sensors used to measure extracellular forces are based on a polyethylene glycol (PEG)-spring. In the molecular tension–based fluorescence microscopy (MTFM) approach, organic dye fluorescence rises as the distance to a synthetic quencher^66^ or a gold nanoparticle (AuNP)^41^ increases in response to stretch. (f) The tension gauge tether (TGT) method uses double-stranded DNA fragments, which separate at defined forces *via* unzipping (low force) or shearing (high force)^76^

A number of approaches to measure molecular tension across cell-surface or intracellular molecules have been developed; they are based upon Förster resonance energy transfer (FRET), photo-quenching, loss of fluorescence or changes in fluorophore emission properties. For a better understanding how to design new biosensors, we will briefly introduce FRET and how it can be measured in cells, before we provide a short overview of the different techniques and their applications (see also Tables 1, 2; Fig. 2).

**Table 1:** Genetically-encoded tension sensors

Name	Sensing element	Sensor sensitivity	Molecular size	Original publication	Application by original group	Independent application
PriSSM	AS(GGS)_9_	pN range	~55 kDa	Iwai *et al.* 29		
stFRET	α-helix	pN range	~56 kDa^a^	Meng *et al.* 46	Meng *et al.* 45	
TSMod	Flagelliform (GPGGA)_8_	1–6 pN; by single-molecule spectroscopy	~56 kDa	Grashoff *et al.* 23	Conway *et al.* 12 Kuriyama *et al.* 35 Leerberg *et al.* 39	Borghi *et al.* 5 Chang *et al.* 9 Cai *et al.* 6 Krieg *et al.* 34
sstFRET	Spectrin repeat^b^	5–7 pN; by DNA springs	~65 kDa	Meng and Sachs^43^	Rahimzadeh *et al.* 56 Verma *et al.* 74	
cpYFP^c^	Chromophore	pN range	~29 kDa	Ichimura *et al.* 28		
cpstFRET		5–7 pN; by DNA springs	~54 kDa	Meng and Sachs^44^		

^a^ The original publication indicates a size of ~70 kDa; based on the used amino acid sequence, however, a size of ~56 kDa is expected

^b ^Other groups have reported spectrin repeat unfolding at ~20 pN^38^ and 25–35 pN^60^

^c^ This sensor has not been used in cells

**Table 2:** **Synthetic tension sensors**

Name	Sensing element	Sensor sensitivity	Sensor calibration	Principle	Original publication	Application by original group
MTFM	PEG_n_^a^	0–20 pN^a^	Theoretical; WLC model^c^	QSF 21-quenching	Stabley *et al.* 66	Jurchencko *et al.* 31
AuNP-MTFM	PEG_n_^a^	0–25 pN^a^	Theoretical; WLC model^c^	AuNP-quenching	Liu *et al.* 41	
MTS	Flagelliform (GPGGA)_8_	1–6 pN	Grashoff *et al.* 23	FRET	Morimatsu *et al.* 47	
TGT	dsDNA tether	12–56 pN	Single molecule AFM	Fluorescence loss^b^	Wang and Ha^76^	

^a ^Force sensitivity can be tuned by adjusting the PEG polymer length

^b^ The TGT response is non-reversible

^c^ Worm-like chain model

### A Brief Introduction to Förster Resonance Energy Transfer (FRET)

FRET is a process in which energy is transferred nonradiatively from an electronically excited donor (D) chromophore to a nearby acceptor (A). The FRET efficiency *E*, defined as the proportion of donor molecules that transfer excitation energy to the acceptor, is highly dependent on the D–A separation distance *r* and characterized by the Förster distance *R*_*0*_.

1 $$E = \frac{{R_{0}^{6} }}{{R_{0}^{6} + r^{6} }}$$

*R*_*0*_ embodies the relative orientation of donor and acceptor dipoles *κ* ^2^, the refractive index *n*, the donor quantum yield *Q*_D_, and the overlap integral of donor emission and acceptor absorption spectra *J*.

2 $$R_{0}^{6} \sim \kappa^{2} n^{ - 4} Q_{\text{D}} J$$

The spectral overlap integral in turn depends on the acceptor extinction coefficient ε_A_ according to

3 $$J\left( \lambda \right) = \int {\varepsilon_{\text{A}} \left( \lambda \right)\lambda^{4} F_{\text{D}} \left( \lambda \right){\text{d}}\lambda }$$

where *F*_D_ is the donor emission spectrum and *λ* the wavelength. Thus, FRET is highly distance-dependent but can be strongly affected by the D–A orientation as well. It is worth noting that the orientation factor *κ* ^2^ is often assumed to be constant throughout the experiment, which may not always be a valid assumption.^36^ In fact, the relative orientation of donor and acceptor transition dipoles has been utilized in orientation-dependent FRET biosensors^44^ (Fig. 2b). The equations above also show that properties of FRET-based biosensors can be adjusted to some degree by employing different donor and acceptor fluorophores with varying quantum yields and extinction coefficients.^63^,^64^ For a more detailed overview of FRET we refer to excellent literature.^30^,^36^

### FRET Measurements in Cells

To fully harness the power of FRET-based biosensors, suitable microscopy techniques and data analysis algorithms are critical. For this purpose, a number of approaches to determine FRET in cells are available.^40^,^75^ One of the most frequently used methods is based on intensity measurements, in which the donor fluorophore is excited and the emission intensities of donor and acceptor fluorophore are used to calculate a FRET ratio. This estimate of relative FRET is useful for biosensors that are characterized by fixed donor/acceptor stoichiometry and can be measured with any appropriately equipped wide-field or confocal microscope. However, these intensity-based measurements do not readily yield quantitative information on FRET efficiencies, are sensitive to the experimental settings (e.g. excitation intensity or biosensor expression level) and require careful image data analysis to account for spectral bleed-through, cross-excitation or photobleaching.^75^ Alternatively, fluorescence lifetime imaging microscopy (FLIM)^48^,^68^,^69^ can be used to calculate FRET efficiencies from the donor lifetime (*τ*) in the presence (DA) or absence (D) of the acceptor.

4 $$E = 1 - \frac{{\tau_{\text{DA}} }}{{\tau_{\text{D}} }}$$

The FLIM approach is insensitive to fluorophore concentration and experimental settings but nevertheless requires rigorous controls and careful data analysis.^75^ Other imaging methods include acceptor photobleaching^73^ or anisotropy measurements,^40^ each with its own advantages and disadvantages. In general, life-cell FRET experiments are complicated by cellular auto-fluorescence, undesired photobleaching and the fact that fluorophore properties depend on environmental factors such as pH, ion concentration or temperature.^52^,^61^ Thus, an in-depth understanding of the limitations inherent to the different FRET analysis methods is essential.^30^,^53^,^69^,^78^

### Genetically-Encoded Tension Sensor Modules for Measuring Intracellular Molecular Forces

Most of the existing tension sensor modules are based upon the initial observation that elastic molecules such as single-stranded DNA (ssDNA) can act as pN force sensors when inserted between two fluorescent dyes undergoing efficient FRET.^65^ Since the FRET efficiency inversely correlates with the D–A distance (Eq. 1), forces that extend the linker and thereby increase chromophore separation strongly reduce FRET (Fig. 2a). Therefore, the selection of an appropriate elastic element is critical and the following requirements have to be satisfied. First, the linker has to be short because currently available FRET pairs are characterized by a Förster distance *R*_0_ ≈ 5–6 nm, at which the FRET efficiency is most sensitive to changes in fluorophore separation distance (Fig. 2a).^53^,^69^ Second, the increase in linker length has to be sufficiently large so that applied tension translates into measurable FRET efficiency differences. Finally, data interpretation is greatly facilitated if the linker follows a simple folding/unfolding pathway and quickly returns to its original conformation when forces subside.

Following these principles, a number of FRET-based tension-sensitive modules have been developed (Table 1; Fig. 2). The linker elements range from a comparably stiff α-helix^46^ and spectrin repeat^43^ to the elastic spider silk flagelliform peptide^23^ (Fig. 2a). An alternative approach was recently tested, in which the force-sensitive element does not change its length but rather conformation (Fig. 2b).^44^ In addition to the FRET-based approaches, a circularly permuted (cp) YFP has been generated that loses fluorescence under force^28^ (Fig. 2c). Similarly, proximity imaging (PRIM) has been used to correlate molecular strain with changes in the emission spectrum of an engineered GFP-dimer^29^ (Fig. 2d). Whether all these techniques will be useful for further applications in cells, however, remains to be determined.

### Synthetic Tension Sensing Techniques for Measuring Forces at the Cell Surface

Measuring mechanical forces at the cell surface does not require genetic encoding of the tension sensing element but can be performed using mechanically well-described polymers. In addition, organic dyes can be employed which are more photostable than most genetically encoded fluorophores and rarely affect the functionality of the labeled molecules. Together with the versatile surface chemistry technologies that are available, these tools have enabled the development of highly sensitive methods to determine extracellular molecular forces (Table 2). For example, the molecular tension–based fluorescence microscopy (MTFM) approach uses polyethylene glycol (PEG) as a force-sensitive tether molecule to measure mechanical tension across growth factor^66^ and cell adhesion receptors^31^,^41^ (Fig. 2e). Similarly, functionalization of the flagelliform peptide^23^ with organic dyes and arginine–glycine–glutamine (RGD)-ligands allows the estimation of force across single integrin receptors^47^ (Fig. 2a). An addition to these synthetic sensors is the tension gauge tether (TGT) approach, where immobilized double-stranded DNA (dsDNA) is functionalized with cell surface receptor ligands so that force above a well-defined threshold can be easily detected^76^ (Fig. 2f).

### Applications of FRET-Based Molecular Tension Sensors

The genetic tension sensor modules described above have been applied to a range of proteins (Fig. 2; Table 1) in different cell types and even whole organisms such as *C.* *elegans* 34 or *D. melanogaster.* 6 The targeted molecules include actin-binding proteins such as α-actinin,^43^–^46^,^74^ filamin,^45^,^46^ and spectrin^34^,^43^,^46^ as well as cell adhesion molecules like cadherin,^5^,^6^,^12^ PECAM-1,^12^ and vinculin.^23^ These measurements confirmed the long held assumption that many cytoskeletal proteins bear pN forces and are an ideal starting point for a more detailed analysis. The use of a vinculin tension sensor, for instance, revealed an average force of about 2.5 pN across vinculin. More interestingly, however, the vinculin transduced tension strongly depends on the cell adhesion state, with highest tension occurring in assembling focal adhesions but low forces in disassembling complexes^23^; this indicates that vinculin stabilizes cell adhesions under mechanical force. In another study, a β-spectrin tension sensor revealed constitutive tension of about 1.5 pN across this cytoskeletal adaptor protein. Interestingly, genetic manipulations decreasing β-spectrin pre-stress correlate with impaired touch sensation suggesting that cytoskeletal pre-tension is critical for efficient mechanosensation in neurons.^34^ These examples illustrate that the true power of FRET-based tension sensors lies not only in the force measurement itself but also in the possibility to unravel molecular mechanisms that are currently inaccessible to other techniques.

## A Guide to Evaluating Genetically-Encoded FRET-Based Tension Sensors

A detailed understanding of a tension sensor’s biophysical properties is crucial. In which force range is the tension sensor module applicable? How does the linker unfold in response to force, and how large is the dynamic FRET range? These kind of questions need to be answered before meaningful experiments can be performed. Furthermore, effects of tension sensor module integration into the protein of interest (POI) need to be carefully evaluated and the FRET experiments must be properly controlled. While every novel genetically-encoded biosensor will require its specific evaluation strategy, we propose here a series of experimental controls which, in our opinion, are indispensable for any FRET-based tension sensor characterization.

### Tension Sensor Design: Which Forces are to be Measured?

Before the experiment, a number of obvious (but not trivial) questions should be addressed. Which molecule should be targeted, what are the expected mechanical forces and do tension sensor modules that are sensitive to these forces exist? It is important to note that molecular tension sensors are unsuitable to measure forces across subcellular structures in general (in fact, this is precisely what they do *not* do), but specifically report tension across the POI. Our previously published vinculin tension sensor, for instance, can be efficiently used to determine vinculin tension but is unsuitable to measure focal adhesion forces in general.^23^ So, it is also worth asking: Are we interested in forces across distinct proteins or across whole subcellular structures?

Once a target protein has been identified, it is necessary to carefully evaluate whether the tension sensor module can be inserted into the POI without significantly affecting its function. We find that structural information is often helpful to identify possible insertion sites, which are preferably unstructured and flexible. In case of the vinculin tension sensor, for example, the chosen integration site is located in a flexible linker region between two well-defined structural domains and vinculin function is preserved after tension sensor module integration.^10^,^23^ If little structure information is available for the POI, we recommend testing several integrations in parallel.

### Characterizing the Tension Sensor Module: What is the Sensor’s Force Sensitivity?

**Figure 3:**
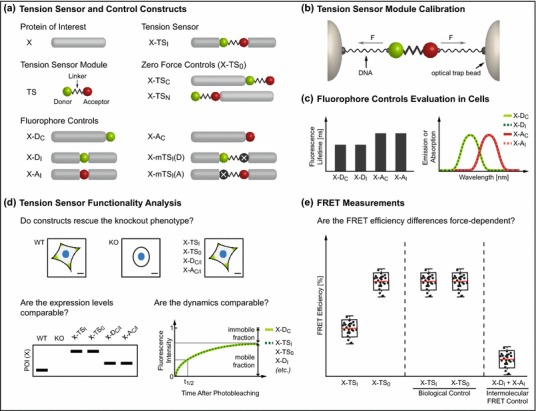
Recommended control constructs and experimental strategies for FRET-based tension sensor evaluation. (**a**) The tension sensor (X-TS_I_) consists of the tension sensor module (TS) internally integrated into the protein of interest (POI) (X). As a zero-force control (X-TS_0_), the TS can be fused C- (X-TS_C_) or N-terminally (X-TS_N_) to the POI. To evaluate functionality of the targeted protein as well as the fluorophores, N- or C-terminal (X-D_C_, X-A_C_) fusions and internal (X-D_I_, X-A_I_) integrations of donor (D) and acceptor (A) fluorophores are recommended. Additionally, tension sensor constructs with non-fluorescent mutant donor (X-mTS_I_(A)) or acceptor fluorophore (X-mTS_I_(D)) can be used. (b) Single-molecule force spectroscopy techniques can be used to calibrate new tension sensor modules. The protein is attached to micro-beads *via* dsDNA handles and an optical tweezer applies pN forces. (c) Fluorescence lifetimes or emission spectra of donor (X-D_C_ vs. X-D_I_ or X-mTS_I_(D)) and acceptor (X-A_C_ vs. X-A_I_ or X-mTS_I_(A)) fluorophores can be compared to test whether fluorophore properties are preserved after insertion into the target protein. (d) Functionality of the tension sensor can be efficiently analyzed by comparing knockout (KO) cell lines reconstituted with the either the tension sensor or control constructs. Ideally, reconstituted cells resemble the parental wild type (WT) cells. Subcellular localization can be checked by fluorescence microscopy; physiological expression levels should be confirmed by western blotting. Subcellular dynamics may be evaluated through fluorescence recovery after photobleaching (FRAP) experiments, which allow the analysis of mobile and immobile fractions. (e) Calculation of FRET efficiencies is recommended to quantify FRET measurements. In addition to genetic controls, where X-TS_I_ is compared to X-TS_0_, biological controls should be included. For instance, chemical inhibitor treatments can be used to prevent force generation across the POI, which should lead to a substantial increase in FRET efficiency of X-TS_I_. Intermolecular FRET can be determined using co-expressed X-D_I_ and X-A_I_ or X-mTS_I_(D) and X-mTS_I_(A)

As discussed above, proteins are subject to a range of pN forces. As the main purpose of a tension sensor is the quantification of these forces, a careful evaluation of the probe’s force sensitivity is required. For elastic elements such as PEG, ssDNA or unstructured polypeptides like (GGS)_*n*_, which are well-described by established polymer models, a theoretical calibration may be sufficient.^65^,^66^ However, experimental calibration is inevitable when more complex linker elements are employed. For such measurements, we strongly recommend the use of single-molecule techniques that allow well-controlled and repeated stretching of sensor peptides over a wide range of forces.^81^ Such a single-molecule calibration has been successfully used to determine the force sensitivity of the flagelliform peptide,^23^ but can also be employed to investigate the force response of a complete tension sensor module including donor and acceptor fluorophores (Fig. 3b) (unpublished observation, *C.* *Grashoff* and *M.* *Rief*). A tension sensor module calibration using optical tweezers typically involves purification of the protein from bacteria or eukaryotic cells, followed by its functionalization and linkage to DNA handles, which are then attached to micro-beads. Application of pN forces by an optical trap allows a detailed analysis of tension sensor module unfolding under force and, importantly, refolding when tension is reduced.

### Biosensor Characterization I: Are the Fluorophores Functional After Integration into the POI?

Next to the *in vitro* calibration of the tension sensor module, its functionality after integration into the POI needs to be validated. Steric constraints, for instance, could impair fluorophore folding. Furthermore, forces of about 35 pN^17^ are sufficient to partially unfold GFP-like proteins, which might influence fluorescence.^61^ Therefore, we recommend comparing the properties of individual donor (D) and acceptor (A) fluorophores terminally fused to the target protein (X) (Fig. 3a, X-D_C_ or X-A_C_) with fluorophores that have been integrated into the POI (Fig. 3a, X-D_I_, X-A_I_). Alternatively, integrated tension sensor modules harboring one non-fluorescent mutant fluorophore (Fig. 3a, X-mTS_I_(D), X-mTS_I_(A)) may be used for a comparison. Fluorescence lifetime as well as absorption or emission spectra are useful parameters to determine whether properties of internally placed fluorophores are affected (Fig. 3c).

### Biosensor Characterization II: Is the POI Functional After Tension Sensor Module Integration?

A critical step in the development of a genetically-encoded biosensor is the insertion of the tension sensor module into the POI; quite obviously, this involves the risk of altering the target protein’s function. Therefore, a detailed evaluation of the biosensor is critical and requires the generation of genetic control constructs (Fig. 3a), for which protocols have been described before.^2^ To evaluate the biosensor’s biological functionality, these constructs should be expressed in cells depleted of the endogenous protein, which has several advantages (Fig. 3d). First, overexpression artifacts can be avoided by adjusting biosensor expression to physiological levels. Second, it can be easily tested whether the biosensor is able to functionally replace the endogenous protein. The β-spectrin tension sensor, for example, rescues the paralysis phenotype of spectrin mutant *C. elegans* to wild type behavior^34^ and an E-cadherin tension sensor was shown to rescue the migration defect in E-cadherin–depleted border cells in *D. melanogaster.* 6 Finally, force measurements are likely to be more accurate as the total amount of force distributes only across biosensor molecules.

A typical evaluation experiment includes the reconstitution of knockout (or knockdown) cells with the tension sensor construct (Fig. 3a, X-TS_I_) and the N- or C-terminally tagged POI (Fig. 3a, X-D_C_ or X-A_C_). This is followed by confirmation of proper subcellular localization using fluorescence microscopy methods as well as the evaluation of expression levels by western blotting. Depending on the POI, functionality may be further tested by fluorescence recovery after photobleaching (FRAP) analysis, where the subcellular dynamics of X-TS_I_ and X-D_C_ can be easily compared (Fig. 3d).

### Controlling the FRET Experiment: Are Effects of Intermolecular FRET or conformation changes significant?

As described above, FRET experiments are complex because energy transfer does not only depend on the chromophore separation distance and orientation but also on the biophysical properties of the individual fluorophores (Eqs. 2 and 3).^53^ Therefore, FRET-based tension sensor experiments need to be carefully controlled.

To ensure that differences in FRET are caused by mechanical tension across the biosensor and are not a result of changes in the microenvironment (such as pH, temperature, etc.), we emphasize the need to use a zero-force control, which can be easily generated by fusing the tension sensor module to either end of the POI (Fig. 3a, X-TS_0_). This control should show identical subcellular localization as the biosensor (X-TS_I_) but should not display changes in FRET as no significant tension can be applied across the module. The second possibly confounding factor in a tension sensor FRET experiment is energy transfer between adjacent molecules (so-called intermolecular FRET) that can significantly contribute to the overall FRET in compact subcellular structures such as focal adhesions or cell–cell contacts. Intermolecular FRET can be easily estimated using a pair of control constructs in which either the individual fluorophores^23^ (Fig. 3a, X-D_I_, X-A_I_) or tension sensor modules with one non-fluorescent mutant fluorophore^12^ (Fig. 3a, X-mTS_I_(D), X-mTS_I_(A)) are integrated into the POI. Co-expression of such constructs in one cell and subsequent FRET measurement in the relevant subcellular structure allow calculation of intermolecular FRET. Furthermore, potential effects of protein conformation changes on FRET need to be considered. As this strongly depends on the molecule of interest, however, these control experiments are not generalizable. Nevertheless, conformation controls should be included to ensure that changes in FRET are reflective of differences in mechanical tension and not *κ* ^2^ artifacts. Finally, the notion that FRET changes actually reflect changes in tension may be reinforced by experiments in which external forces are rapidly applied using mechanical stretch^5^ or fluid shear flow.^12^

### Data Analysis and Interpretation: What Do the FRET Efficiency Differences Mean?

At the end, proper data analysis is critical. We highly recommend the use of quantitative techniques such as fluorescence lifetime imaging microscopy (FLIM) allowing the calculation of FRET efficiencies instead of FRET ratios (Fig. 3e). Moreover, automated data analysis software to determine transfer rates in subcellular compartments greatly facilitates data interpretation. The evaluation experiments described above should be followed by additional controls that will depend on the individual context (such as inhibition of intracellular contractility or application of external forces). Together, this experimental strategy will allow a straightforward evaluation of new tension-sensitive biosensors. We wish to emphasize that insufficiently characterized tension sensors should not be utilized by the scientific community as their application all too often results in misleading interpretations and confusion.

## Outlook

While the development of molecular tension sensors has already made significant contributions to a deeper understanding of force transduction, further improvements will be necessary to further elucidate molecular mechanisms. For instance, more calibrated tension sensor modules are required to evaluate distinct force ranges. Also, probes with increased dynamic range, quantum yield and photostability would be useful to perform intracellular single-molecule measurements that unravel the heterogeneity and dynamics of molecular processes. In this context, the development of orthogonal labeling techniques using genetically encoded proteins,^13^ peptides, and non-natural amino acids^37^ is promising, as they allow site-specific labeling of intracellular proteins with organic dyes. Another approach that seems worth pursuing is genomic integration of biosensors into the locus of the target proteins by the recently developed CRISPR/Cas9 technique.^62^ This strategy should ensure physiological expression levels of a biosensor and avoid the time-consuming generation of knockout (or knockdown) cell lines. Finally, other approaches to determine forces in cells could be combined with FRET-based tension sensors. These may include optical tweezers methods that can be applied to individual molecules within cells,^49^ the specific functionalization of micro-droplets, which were recently used to determine mechanical forces on the cellular level in embryonic tissue,^7^ or traction force microscopy techniques allowing the simultaneous measurement of traction forces and molecular forces in cell adhesions. In fact, while this manuscript was under revision, two new, synthetic tension sensor techniques that use hairpin-DNA as force sensitive linkers and fluorescence quenching as read-out were published,^4^,^79^ which allow molecular traction force microscopy.

In summary, properly characterized molecular tension sensors provide a powerful tool to gain insight into cellular mechanotransduction. Further improvements that will allow experiments at single-molecule resolution within cells, the application of tension sensors to a wider range of proteins, and the combination of biosensors with other quantitative techniques may pave the way to a better understanding of how cells sense and respond to their mechanical environment.
